# Cluster of systemic lupus erythematosus (SLE) associated with an oil field waste site: a cross sectional study

**DOI:** 10.1186/1476-069X-6-8

**Published:** 2007-02-22

**Authors:** James Dahlgren, Harpreet Takhar, Pamela Anderson-Mahoney, Jenny Kotlerman, Jim Tarr, Raphael Warshaw

**Affiliations:** 1 Department of Occupational and Environmental Medicine, UCLA School of Medicine, Los Angeles, CA, USA; 2 James Dahlgren Medical, Santa Monica, CA, USA; 3 Epidemiology Resources, Van Nuys, CA, USA; 4 Epidemiology, UCLA School of Public Health, Los Angeles, CA, USA; 5 Stone Lions, Rolling Hills Estates, CA, USA; 6 Comprehensive Health Screening Services, Santa Monica, CA, USA

## Abstract

**Background:**

This is a community comparison study that examines persons living in a subdivision exposed to petroleum products and mercury.

**Methods:**

We compared their health status and questionnaire responses to those living in another community with no known exposures of this type.

**Results:**

Pristane house dust among the exposed homes was higher than in the comparison communities. The exposed subdivision has higher ambient air mercury levels compared to the control community. The prevalence of rheumatic diseases (OR = 10.78; CI = 4.14, 28.12) and lupus (OR = 19.33; CI = 1.96, 190.72) was greater in the exposed population compared to the unexposed. A higher prevalence of neurological symptoms, respiratory symptoms and several cardiovascular problems including stroke (OR = 15.41; CI = 0.78, 304.68) and angina (OR = 5.72; CI = 1.68, 19.43) was seen.

**Conclusion:**

There were statistically significant differences in B cells, Natural Killer Cells, gamma glutamyl transferase, globulin and serum calcium levels between control and exposed subjects.

## Background

**Figure 1:**
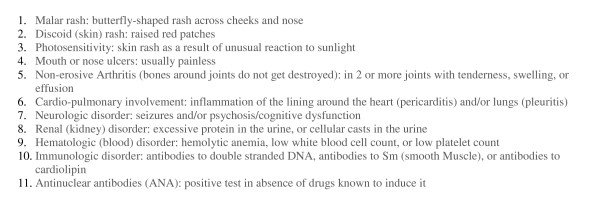
American College of Rheumatology – The "Eleven Criteria" for diagnosis of SLE.

Systemic lupus erythematosus (SLE or lupus) is an autoimmune disease in which the body produces anti-nuclear antibodies that attack healthy tissues leading to inflammation and damage to various body tissues. Lupus can affect many parts of the body, including the joints, skin, kidneys, heart, lungs, blood vessels, and brain. It is a chronic, complex, and potentially fatal multi-system inflammatory disorder that can be difficult to diagnose [1,2]. No single laboratory test confirms a diagnosis of SLE. Many physicians use the American College of Rheumatology's "Eleven Criteria of Lupus" to aid in the diagnosis where the appearance of four of the "Eleven Criteria of Lupus" qualifies as a positive diagnosis of Lupus [2] (Figure 1).

SLE can occur at any age and in either sex. However, women are more likely to have SLE and women of color are more likely to have SLE compared to white women [3]. Migration studies suggest that environmental factors play a role in the development of SLE [4]. Residents living near industrial emissions or environmental contamination have been shown to have an increase prevalence of SLE [5,6]. Research indicates that a combination of genetic and environmental factors can trigger the development of SLE, however there is a need for additional research to identify and characterize the specific exposures that contribute to the incidence and aggravation of SLE [7,8].

Animal studies suggest that pristane and mercury may be environmental triggers for SLE [9-15]. It has been demonstrated that both pristane and mercury will induce a condition in mice that mimics clinical features and the autoantibody pattern characteristic of SLE in humans. We know of no reports in the medical literature of SLE in humans following exposure to pristane. There have been case reports linking mercury to autoimmune disease in humans and a recent epidemiologic study of occupational risk factors for SLE identified mercury as a potential causal agent (OR = 3.6; 95% CI = 1.3,10.0) [7-10].

We investigated an apparent cluster of SLE cases in a community in Hobbs, New Mexico. This investigation was initiated by residents of a six square block area who noted an excess of SLE cases in their neighborhood. Most of the cases occurred in a new subdivision built on land that was an active oilfield from 1927 until the late 1960s. This subdivision was built on that site in 1976. Some of the homes were built on ground that had previously been used as a pit for oil field waste, which was estimated to be 200 feet long and 30 feet wide.

The residents experienced petroleum and/or rotten egg odors inside their homes on frequent occasions. They also found black oily material oozing out of the ground either spontaneously or when digging in the soil around their property. The residents sought legal advice because they were concerned that there was a connection between the apparent residual oil field waste and the elevated SLE cluster in their neighborhood.

**Figure 2:**
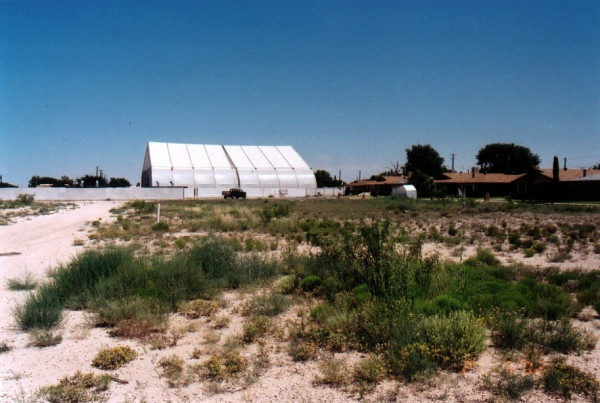
Photograph of Oil Field in Relation to Residents Homes.

**Figure 3:**
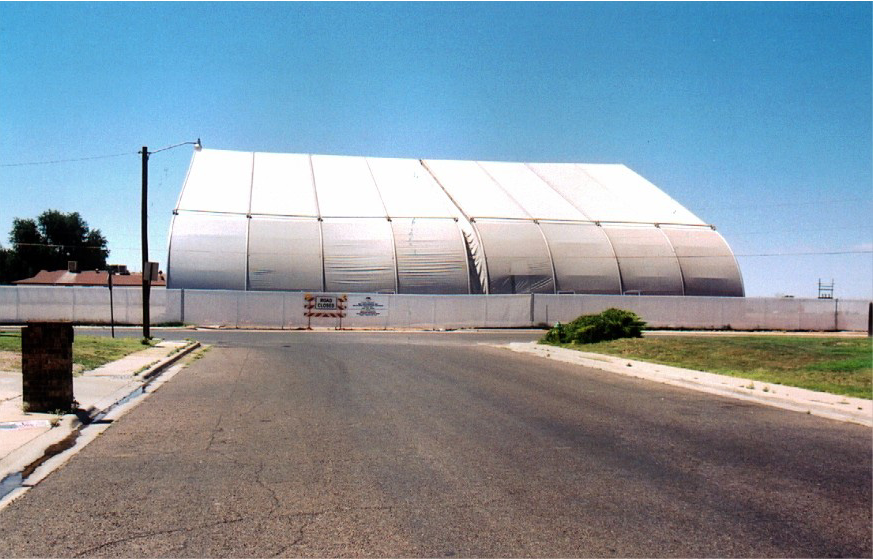
Close up Photograph of Tank Battery in Relation to one of the two exposed Streets.

There was a tank battery and several active oil wells located directly to the west of the subdivision which continued operating until 2000 (Figures 2 &3). The oil company had installed a vapor recovery system for these oil wells and an acompanying tank battery to reduce vapors escaping from the storage tanks in 1969. When a lawsuit was filed the oil company investigated the area and based on the results of their investigation, closed down the tank battery and purchased the three homes closest to that tank battery site. The surface soil from the tank battery and home sites was transferred to a hazardous waste site because of very high Total Petroleum Hydrocarbons (TPH). Soil testing for metals, semi-volatiles and polycyclic aromatic hydrocarbons (PAHs) at other nearby homes did not reveal levels high enough to oblige remediation. Both soil and air testing by the oil company and the experts retained by the plaintiffs' counsel revealed the presence of aromatic hydrocarbons including benzene, toluene, ethylbenzene, xylene, pristane and phytane. People are still living in the remaining adjacent homes. We compared the health status of 90 residents along with their environmental and biomonitoring test results to a reference population.

## Methods

### Study design

This is a community comparison study that examines persons living in a subdivision exposed to petroleum products and mercury and compares their health status and questionnaire responses to those living in another community with no known exposures of this type. A volunteer sample of 90 adults from the exposed neighborhood completed a questionnaire and donated blood for the measurement of pristane, pristanic acid and phytane. We compared the environmental exposures and questionnaire responses and pristane/phytane blood levels to those living in another community with no unusual exposures to these contaminants. We compared the observed prevalence of SLE in this community with values reported in the literature. Exposed study participants were all plaintiffs in a lawsuit.

### Setting and study populations

Exposed Population – Hobbs, New Mexico is a predominantly Caucasian (63.5%) town of 28,657 residents located in Lea County on the southeast corner of New Mexico, 5 miles from the Texas border [16]. Hobbs was founded in 1907 as an agricultural and ranching community and became prominent after the discovery of oil in 1928. Hobbs is known as the oil capital of New Mexico [17]. Numerous oil and gas wells are scattered throughout the area and this industry is the principal source of employment in Hobbs. We estimated a total population of 1490 residents in the study neighborhood by counting 532 homes and estimating 2.8 individuals in each household. The bulk of the SLE cases are on two streets that roughly correspond to the location of the oil field waste pit until it was covered with fill dirt in the late 1960's. The study population of 90 adults had lived in the area for at least two years, and voluntarily enrolled in the study. We assumed that the rest of community did not have SLE.

Comparison Population – One hundred and twenty nine volunteers from a similar southwestern town without unusual chemical exposures were recruited through a church. We invited the members to participate in the study by filling out a questionnaire and volunteering to have blood drawn for biomonitoring. As with the exposed population, trained and experienced proctors administered a nearly identical questionnaire to all volunteers in small groups. The questionnaire differed only with respect to questions regarding exposure experiences unique to the Hobbs neighborhood. The control subjects were paid a small fee for their participation. The control town was matched for size, altitude, and demographics. The control population was not free of unusual petroleum hydrocarbon exposure. Fifteen of the controls had been raised in Bakersfield a town similar to Hobbs with many nearby oil fields. Furthermore, the town is the site of a large railroad-switching yard. Given that Hobbs has a large Hispanic population we note some possible dietary issues specific to Hispanic populations (herbal teas, etc.).

### Data collection

The questionnaire obtains data on demographics including age, gender, occupational and residential history as well as medical, social and behavioral history. Other topics covered in the questionnaire are health symptoms, diseases, surgeries, medications, family history, income, chemical exposures, and life style measures including smoking and alcohol drinking. One unlikely symptom question is designed to test for the veracity of the responses provided. Questionnaire responses are machine-readable, scanned on-site and verified before subjects leave. This basic questionnaire has been used in prior studies of exposed and unexposed groups [18].

### Case definition

We defined a case of SLE as an individual who had received a physician's diagnosis. We confirmed the diagnosis with medical records to confirm that the diagnosis had been reached in accordance with American Rheumatology Association's "Eleven Criteria of Lupus" [12]. We excluded cases that were diagnosed within 6 months of moving to the neighborhood or cases that were diagnosed more than 5 years after moving away. This criteria for diagnosis is consistent with previously published studies [5,19].

### Exposure assessment

#### House dust

We collected house dust samples from residents who permitted access to their homes. House dust samples were collected in the exposed and control community from 2/27/03 to 3/1/03 by Stone Lions Environmental Corporation (Rolling Hills Estates, CA). A total of 19 house dust samples were collected in the exposed subdivision and three additional samples were taken about 2 miles northeast of the subdivision. Nine house dust samples were collected from the control community.

Stone Lions Environmental Corporation collected house dust samples using current state-of-the-art method for household dust sampling which involves using the HVS-3 forensic vacuum or a Sears Kenmore canister vacuum model 22085 sampling system. Dust was drawn into a new vacuum bag, which was removed after each house. The vacuum bags were immediately placed into a double Ziploc bag and labeled accordingly. Samples were collected in various places in each house depending on the availability of dust. The primary locations were attic, heater vents, windowsills, tops of furniture and appliances, tops of doorways and doorway frames, exposed shelves, and carpet (only for houses with minimal dust elsewhere). All cleaning and bag removal activities were performed while wearing powder free surgical gloves. The samples were analyzed for analytes, polycyclic aromatic hydrocarbons (PAHs), total petroleum hydrocarbons (TPH), radiochemistry, pristane and phytane. Metals, PAHs, and TPH were analyzed by West Coast Analytical Services (Santa Fe Springs, CA). Metals were analyzed using Inductively Coupled Plasma – Mass Spectrometry. PAHs were analyzed using EPA method 625/8270C/SIM. TPH were analyzed using EPA method 418.1. Radiochemistry was analyzed at Fruit Growers Laboratory (Santa Paula, CA) using 901.0 (Gamma isotopic and 9310 (Radiochemistry). Pristane and phytane exposures were analyzed at Humble Geochemical Services (Humble, Texas) using high-resolution gas chromatography. This method to measure and quantify pristane and phytane in crude oil is standard in the petroleum industry. The petroleum industry uses pytane/pristane fingerprints to determine the source of crude oil.

#### Air monitoring

An ambient air monitoring station for volatiles and reduced sulfur compounds was established at a site located directly on the old waste pit in the exposed subdivision. The site was located on a front lawn within a 10 by 10 foot chain link fence. Silica-lined Summa canisters were used to collect 24 ambient air samples on a schedule of approximately once every six days. The first sample was collected on October 18^th^, 2002 and the final sample was collected on February 11^th^, 2003. Over that period of five months, nineteen 24-hour samples were collected including one field blank. Canister preparation and sample analyses were performed by Zymax Envirotechnology (San Luis Obispo, California). Each sample was analyzed for volatile organic compounds (VOCs) and reduced sulfur compounds. VOCs were analyzed using EPA method TO-14 GC/FPD.

A meteorological station (Davis Instruments) was installed at the same location as the ambient air-monitoring site. Instruments measuring wind speed, wind direction, ambient temperature, pressure, relative humidity and rainfall were mounted on a two-meter tower. Those parameters were recorded at half-hour intervals for the duration of the ambient air-monitoring period.

#### Mercury ambient air testing

The Lumex Zeeman Mercury Analyzer RA-915+ was used to measure the ambient air concentration of mercury from various locations inside and outside the homes in both the exposed and control communities. 30-second ambient air samples were taken in the center of each room and on the front porch.

### Biomonitoring

#### General health screening panel

A trained phlebotomist collected blood and urine from volunteers from both the exposed and control communities. One tiger top, two lavender tops and one grey top (for urine) was shipped overnight on ice to Pacific Toxicology Laboratories (Woodland Hills, California) for analyses. A complete blood count, chemistry panel and a urinalysis were performed using standard laboratory techniques.

#### Lymphocyte subpopulation analysis

The Lymphocyte Subpopulation Analysis (Enumeration Panel) was done to estimate the distribution of the common lymphocytes. A trained phlebotomist collected one yellow top of blood from both exposed and control participants and shipped overnight on ice to Immunoscience Laboratories (Beverly Hills, California)

#### Pristane and phytane

Pristane is a straight chain seventeen carbon alkane; phytane is an eighteen-carbon alkane. Pristanic acid is a metabolite of pristane. All three were measured in blood of exposed and comparison subjects by Southwest Research Institute (San Antonio, Texas). An aliquot of 1 ml of serum was removed and 20 μl of phosphoric acid was added to the serum sample. Pristanic acid-d3 was then added to the serum to monitor the extraction efficiency of pristanic acid. The serum was extracted twice using 5 ml hexane saturated with acetonitrile. The organic layer was decanted and was concentrated to 1 ml. The organic extract was then derivatized using diazomethane to convert pristanic acid to its ester form. After derivatization, the organic extract was further concentrated to 0.2 ml and serum samples were ready for GC/MS analysis. The GC/MD instrument was calibrated using a 5-pt calibration curve. The range was from 0.8 – 0.25 ug/ml. The instrument was operated under selected ion monitoring (SIM) mode to enhance sensitivity.

### Data analysis

Unadjusted frequencies and percents for age, gender, ethnicity, smoking status and education are presented for exposed and unexposed populations. P-values for the unadjusted differences in percents between the two groups were estimated using the Pearson chi square test. Odds ratios and 95% confidence intervals are estimated for binary health outcomes using logistic regression to compare exposed and unexposed populations while controlling for age, gender, education and race/ethnicity. The Hosmer and Lemeshow goodness-of-fit test (Hosmer and Lemeshow 1989) for the case of a binary response model was performed for each model. In this procedure, the subjects are divided into approximately ten groups of roughly the same size based on the percentiles of the estimated probabilities. The discrepancies between the observed and expected number of observations in these groups are summarized by the Pearson chi-square statistic, which is then compared to a chi-square distribution with ***t ***degrees of freedom, where ***t ***is the number of groups minus ***n***. By default, ***n ***= 2. A small ***p***-value suggests that the fitted model is not an adequate model.

Odds ratio and confidence intervals are estimated for those health outcomes where the response possibilities include a scale from 1 to 11 using multinomial logistic regression models. The odds ratio is interpreted as the odds of the exposed reporting a higher response from the 1 – 11 scale compared to the unexposed. All the ordinal outcome variables were fit into the ordinal logistic model and the goodness of fit was tested using the chi-square statistic.

All statistical analyses were performed using SAS 8.0.

## Results and discussion

Ninety adult volunteers from the exposed community and 129 adults from the comparison community participated in the study (Table 1). The age, gender and smoking history (ever/never) were similar between the two groups. The exposed population was more diverse in terms of race/ethnicity; the comparison group was Caucasian. Level of education was higher in the comparison group.

**Table 1:** Demographic frequencies in Hobbs* and in a control community**

	**Exposed**		**Unexposed**		
	**No.**	**%**	**No.**	**%**	**P-value**

**Age Category**					
18–34	35	37.63	37	28.68	0.85
35–49	35	37.63	62	48.06	
50–64	19	20.43	28	21.71	
65+	4	4.30	2	1.55	
Total	93		129		
					
**Race/Ethnicity**					
Hispanic	44	48.89	5	3.88	<0.0001
White	29	32.22	121	93.80	
African American	12	13.33	0	0	
Others	5	5.55	3	2.33	
					
**Gender**					
Male	34	37.36	61	47.29	0.21
Female	57	62.64	68	52.71	
					
**Ever Smoked**					
Yes	35	38.89	52	40.31	0.83
No	55	61.11	77	59.69	
					
**Education Level**					
Less than 9^th ^grade	6	6.45	0	0	<0.0001
9 – 11^th ^grade	22	23.66	13	10.08	
12^th^/Vocational/Some College	57	61.29	86	66.67	
College Graduate	8	8.60	30	23.26	

*a residential population exposed to petroleum products and other environmental contaminants

**no known exposures.

### Environmental measures

House dust samples for pristane and phytane were higher in the exposed homes (Table 2). Pristane and phytane were found in every sample tested from both the exposed and unexposed communities, however, significantly higher values were found in the exposed community. Pristane house dust among the exposed homes was higher than in the comparison communities. House dust samples for mercury were not elevated in exposed homes (data not shown).

**Table 2:** Results for pristane and phytane house-dust sampling for Hobbs, Hobbs-Control, and Control Community.

Sample Group/Chemical	Pristane*				Phytane*			
	
	Avg	SD	Max	Min	Avg	SD	Max	Min
Hobbs (n = 19)	480.831	784.216	2441.230	13.770	262.522	542.694	2464.530	18.750
Hobbs-Control** (n = 3)	42.437	25.868	66.290	14.940	155.277	153.763	324.320	23.730
Tehachapi (n = 9)	99.029	116.491	349.310	17.940	223.969	138.587	557.440	85.220

* results in ppm

** Samples collected 2 miles outside of the Westgate Subdivision

AVG – Average

SD – Standard Deviation

Max – Maximum

Min – Minimum

Air sampling by the oil company for pristane and phytane in the exposed neighborhood during both at baseline and soil removal operations consistently showed positive values (Table 3). The baseline air sampling was conducted in July of 2001. The air samples were taken during the remediation that lasted approximately 6 months starting in March of 2002. The SVOC samples were obtained in the exposed subdivision at 6 sites.

**Table 3:** Air monitoring data for pristane, phytane and PAHs taken at baseline and during trenching operations*

	**Summary Statistics for SVOC Air Monitoring – Baseline**			**Summary Statistics for SVOC Analysis of Air – Trenching Operations**		
**Compound**	**Concentration (ng/m**^3^)			**Concentration (ng/m**^3^)		
	
	**Minimum**	**Maximum**	**Mean**	**Minimum**	**Maximum**	**Mean**

Phytane	6.10	145	37.7	7.85	83.9	43.1
Pristane	4.43	182	50.5	8.21	110	41.9
Total PAH	57.0	655	178	89.4	769	224

*Sampling conducted by Oil Company

Results of ambient mercury air measurements are displayed in Table 4. Outdoor air blanks (front porch readings) were consistently much lower than indoor values. Data is not shown. Summary of the ambient mercury air measurements are displayed in Table 5. The exposed subdivision has nearly 6.2 times higher mercury levels compared to other locations in Hobbs located two miles away and nearly 2.5 times higher mercury levels compared to the control community.

**Table 4:** Results for ambient air mercury sampling for Hobbs, Hobbs-Control, and Control Community.

Sampling Group	Address	MULTIPLE SAMPLES AT EACH RESIDENCE*															Average
			
		1	2	3	4	5	6	7	8	9	10	11	12	13	14	15	
Hobbs	Point 1	19	18	21	19	21											19.6
	Point 2	17	18	18	18	20	19	19	18								18.38
	Point 3	7	8	8	10	17	17	15	16	16							12.67
	Point 4	46	73	79	82	68	94	97	157	171	67	103	96	72.3	90	19	87.64
	Point 5	11	15	32	17	34	23	24	28	27	28	20					23.55
	Point 6	11	9	10	9	12	12	11	14	10							10.89
	Point 7	13	14	13	13	15	13	14									13.57
	Point 8	32	24	33	27	30	35	30	32	35							30.89
	Point 9	19	15	14	12	9	15	14	2								12.5
	Point 10	14	13	13	14	10	12	11									12.43
Hobbs Control**	Point 11	7	5	7	6	9	9	7									7.14
	Point 12	4	0	0	0	0	0										0.67
Control	Point 13	15	17.5	15	19	19	21										17.75
	Point 14	2	5	6	6	7											5.2
	Point 15	16	15	16	8	13	28	31	30	29	29	32					22.45
	Point 16	7	9	9	12												9.25
	Point 17	6	7	7	8												7
	Point 18	18	18	18	12												16.5
	Point 19	9	7	6	1	7	5										5.83
	Point 20	4	5	6	3	2	4	9									4.71
	Point 21	10	11	8	16	12	6										10.5
	Point 22	5	6														5.5

* 30-second averages, results in nanograms per cubic meter

**Samples collected 2 miles outside of the Westgate Subdivision

**Table 5:** Summary Results for ambient air mercury sampling for Hobbs, Hobbs-Control, and Control Community.

Sample Group/Chemical	Mercury*			
	
	Avg	SD	Max	Min
Hobbs	24.21	31.34	171.00	2.00
Hobbs-Control**	3.90	3.67	9.00	0.00
Tehachapi	10.47	8.08	32.00	1.00

* 30-second averages, results in nanograms per cubic meter

**Samples collected 2 miles outside of the Westgate Subdivision

AVG – Average

SD – Standard Deviation

### Biomonitoring

One of twenty-five (4.0%) from the comparison subjects and five of twenty (25%) from the exposed who were tested had detectable positive blood levels for pristane, phytane and/or pristanic acid. Each subject who had a detectable level of pristane, phytane and/or pristanic acid also had either a frank diagnosis of lupus or common symptoms associated with immune system disorders. The students' t-test produces a p-value of < 0.05 for this difference.

Blood samples were obtained from 97% (87/90) of the exposed subjects. Forty-three of 90 (47%) exposed subjects and 37/129 (28%) unexposed subjects agreed to provide additional vials of blood for natural killer cell and CD 19 (B cell) analysis. Ten additional control subjects participated only in the natural killer cell and CD 19 (B cell) analysis (Table 6). There were biologically and statistically significant differences in B cells, Natural Killer Cells, gamma glutamyl transferase, globulin and serum calcium levels. Creatinine Phosphokinase (CPK) was not significantly different for the overall group; however, an examination of blood results for men only reveals the mean value in the exposed population is 220 versus 139 in the comparison group. Five of the nineteen exposed males (26.3%) had CPK above the laboratory normal of 269 IU/L and none of the comparison men were above that value. There was no influence by alcohol or other factors.

**Table 6:** Ordinary least squares regression analysis comparing blood results between exposed and unexposed, controlling for age.

**Adults**	**Exposed**		**Unexposed**		**Comparison Estimates**	
	Number	Mean (SD)	Number	Mean (SD)	Parameter Estimate	P-value

% CD 19 (B-cell)	43	18.07(6.11)	47	14.02(4.68)	3.98	0.0006
% Natural Killer Cells^1^	43	10.77(5.65)	47	14.26(7.33)	-3.41	0.01
Total bilirubin	87	0.61(0.23)	37	0.54(0.16)	0.07	0.12
gamma-Glutamyl transferase (IU/L)	87	40.07(50.25)	37	19.30(11.53)	21.60	0.01
Globulin (mg%)	87	3.02(0.50)	37	2.86(0.34)	0.15	0.09
Serum CALCIUM (mg%)	87	9.71(0.38)	37	9.23(0.36)	0.48	<0.0001
Creatinine Phosphokinase (IU/L)	87	127.44(90.63)	37	110.43(54.02)	16.02	0.32

1. Natural Killer Cells = CD16 + 56+/CD45+

SD = Standard Deviation

### Disease prevalence and symptoms

Hosmer and Lemeshow tests were performed on all of the outcomes modeled with logistic regression. All of the p-values are between 0.22 and 0.99. These results indicate that the data fit the model adequately in each case. For data scaled data modeled with multinomial logistic regression, the chi-square test for goodness of fit indicated an appropriate fit for each of the modeled outcomes.

Lupus cases were confirmed by both phone call follow up and review of medical records (Table 7). The prevalence of rheumatic diseases and lupus was greater in the exposed population compared to the unexposed (OR = 10.78; CI = 4.14, 28.12 and 19.33; 1.96, 190.72, respectively) (Table 8). The wide confidence interval for lupus reflects the single case found in the unexposed community. Increased prevalences of symptoms thought to be predictive of autoimmune disorder were found in the exposed community including: numb fingers, mouth sores, and persistent rash on the cheeks and pain on deep breath.

**Table 7:** Exposure information for Lupus cases in Westgate Subdivision

**Patient**	**From Age**	**To Age**	**Total years**	**Gender**	**Date of Birth (age)**
1	43	Present	14	Female	8/21/45 (57)
2	26	Present	22	Female	11/6/54 (48)
3	11	22	11	Female	1/4/76 (27)
4	30	Present	19	Female	1/23/54 (49)
5	37	Present	12	Female	7/3053 (49)
6	31	45	14	Male	6/7/56 (46)
7	33	35	2	Female	8/7/45 (57)
8	38	43	5	Female	12/4/39 (63)
9	28	30	2	Female	1/21/63 (40)
10	62	72	10	Female	12/20/26 (76)
11	18	40	22	Female	4/20/59 (43)
12	35	49	14	Female	9/23/51 (51)
13	3	29	26	Female	12/12/1972 (29)

**Table 8:** Estimated odds ratios and confidence intervals for autoimmune disorders comparing residents to controls

	Exposed N(%) or mean ± s.d.	Unexposed N(%) or mean ± s.d.	Odds Ratio	95% Confidence Interval	P-value
Rheumatic Diseases	33 (39.29)	16 (12.40)	10.78	4.14, 28.12	<0.0001
Systemic lupus erythematosus	13(14.29)	1 (0.78)	19.33	1.96,190.72	0.01
Immune	9 (11.11)	1 (0.78)	26.44	2.67,261.50	0.005
Anemia	22 (26.19)	29 (22.48)	1.14	0.49, 2.65	0.76
Numbness in Fingers	49 (58.33)	34 (26.36)	3.96	1.93, 8.15	<0.0002
Mouth Sores	18 (21.43)	6 (4.65)	5.66	1.78, 18.03	0.03
Rash on the Cheeks	17 (20.24)	4 (3.10)	8.27	2.20, 31.13	0.002
Rash from Sunlight	17 (20.24)	11 (8.53)	1.44	0.50, 4.13	0.50
Pain on Deep Breath	19 (22.62)	7 (5.43)	11.04	3.68, 33.13	<0.0001

*Odds ratio and confidence intervals are estimated using logistic regression

Controlled for age, gender, race/ethnicity, education and smoking history

The presence of neurological symptoms was elevated in the exposed community including: dizziness, lightheadedness, loss of balance, extreme fatigue, sleep disorders, lack of concentration and memory loss (Table 9).

**Table 9:** Odds ratios and confidence intervals for neurologic and behavioral disorders comparing exposed and unexposed residents

	Exposed N(%) or mean ± s.d.	Unexposed N(%) or mean ± s.d.	Odds Ratio	95% Confidence Interval	P-value
Dizziness*	4.40 ± 3.10	2.25 ± 1.96	4.42	2.37, 8.24	<0.0001
Lightheadedness*	4.85 ± 3.12	2.65 ± 1.81	5.02	2.69, 9.34	<0.0001
Loss of balance*	3.68 ± 3.07	2.24 ± 1.73	2.83	1.53, 5.22	0.0009
Extreme fatigue*	6.97 ± 3.69	3.12 ± 2.40	11.50	5.94, 22.28	<0.0001
Somnolence*	5.08 ± 3.76	2.11 ± 1.86	4.99	2.66, 9.36	<0.0001
Can't fall asleep*	4.79 ± 3.79	2.89 ± 2.50	1.94	1.06, 3.54	0.03
Wake up frequently*	5.12 ± 3.79	2.91 ± 2.55	3.98	2.16, 7.36	>0.0001
Sleep soundly for only a few hours*	4.83 ± 3.59	2.81 ± 2.51	4.00	2.16, 7.40	>0.0001
Lack of concentration*	5.70 ± 3.73	3.76 ± 2.66	2.67	1.47, 4.84	0.001
Recent memory loss*	5.22 ± 3.82	3.76 ± 2.66	2.87	1.58, 5.23	0.0006
Decreased libido*	4.36 ± 4.00	2.92 ± 2.35	3.00	1.60, 5.59	0.0006
When driving in familiar areas, do you ever get lost or go the wrong way *	2.67 ± 2.73	1.55 ± 1.33	3.05	1.54, 6.08	0.002

* Odds ratio and confidence intervals are estimated using multinomial logistic regression models with generalized estimating equations (GEE); odds ratio interpreted as the odds of exposed being in a higher category compared to unexposed. Respondents were asked to "score each question on a scale of 1 – 11, one representing never and 11 representing always.

Controlled for age, gender, race/ethnicity, education and smoking history

A higher prevalence of several cardiovascular problems occurred in the exposed population including stroke and angina (OR = 15.41; CI = 0.78, 304.68 and 5.72; 1.68, 19.43, respectively) chest tightness and pain in the chest (Table 10). Again, the wide confidence interval for stroke reflects the paucity of stroke sufferers in the comparison community. No difference was found for the overall measure of heart disease or myocardial infarction.

**Table 10:** Odds ratios and confidence intervals for cardiovascular disorders between exposed and unexposed residents.

	Exposed N(%) or mean ± s.d.	Unexposed N(%) or mean ± s.d.	Odds Ratio	95% Confidence Interval	P-value
Heart Disease*	5 (6.17)	4 (3.10)	1.80	0.33, 9.66	0.50
Acute Myocardial Infarction *	3 (3.57)	3 (2.33)	2.11	0.17, 25.93	0.56
Stroke*	5 (5.95)	1 (0.78)	15.41	0.78, 304.68	0.07
Angina*	15 (17.86)	5 (3.88)	5.72	1.68, 19.43	0.005
Rhythm	10 (12.35)	18 (13.95)	0.61	0.22, 1.68	0.34
Chest tightness**	3.74 ± 2.82	1.92 ± 1.54	5.97	3.13, 11.40	>0.0001
Palpitations**	3.03 ± 2.69	2.35 ± 1.98	1.64	0.87, 3.07	0.12
Pain in Chest**	3.42 ± 2.88	1.73 ± 1.65	4.24	2.21, 8.14	<0.0001

*Odds ratio and confidence intervals are estimated using logistic regression

** Odds ratio and confidence intervals are estimated using multinomial logistic regression models with generalized estimating equations (GEE); odds ratio interpreted as the odds of exposed being in a higher category compared to unexposed. Respondents were asked to "score each question on a scale of 1 – 11, one representing never and 11 representing always.

Controlled for age, gender, race/ethnicity, education and smoking history

Respiratory symptoms were significantly elevated in the exposed population including shortness of breath and wheezing, cough with blood or mucus, dry cough and chronic bronchitis (Table 11).

**Table 11:** Odds ratios and confidence intervals for respiratory disorders between exposed and unexposed residents

	Exposed	Unexposed		95%	
	N(%) or mean ± s.d.	N(%) or mean ± s.d.	Odds Ratio	Confidence Interval	P-value

Pneumonia*	11(13.58)	18(13.95)	1.19	0.45, 3.15	0.72
Pleurisy*	5 (6.33)	4 (3.10)	1.48	0.29, 7.61	0.64
Chronic bronchitis*	16 (19.75)	3 (2.33)	17.4	4.06, 74.35	0.0001
Dry cough**	4.64 ± 3.21	2.36 ± 1.74	5.06	2.67, 9.60	<0.0001
Cough with mucous**	4.73 ± 3.51	2.79 ± 1.75	2.6	1.43, 4.73	0.002
Cough with Blood**	2.30 ± 2.55	1.11 ± 0.45	11.8	4.33, 32.03	<0.0001
Asthma Diagnosed by MD*	11 (13.41)	17 (13.18)	2.13	0.78, 5.82	0.14
Rhinitis*	24 (29.63)	21 (16.28)	3.45	1.49, 8.01	0.004
Sinusitis range**	4.57 ± 3.71	2.50 ± 1.91	2.62	1.43, 4.79	0.002
Short of Breath at Rest*	23 (27.38)	4 (3.10)	11.3	3.11, 40.98	0.0002
Short of Breath on Walking*	40 (48.19)	6 (4.65)	34.3	10.52, 112.09	<0.0001
Short of Breath Climbing Stairs*	52 (62.65)	34 (26.36)	6.17	2.81, 13.56	<0.0001
Wheezing*	24 (28.57)	7 (5.43)	19.2	5.59, 65.98	<0.0001

*Odds ratio and confidence intervals are estimated using logistic regression

** Odds ratio and confidence intervals are estimated using multinomial logistic regression models with generalized estimating equations (GEE); odds ratio interpreted as the odds of exposed being in a higher category compared to unexposed. Respondents were asked to "score each question on a scale of 1 – 11, one representing never and 11 representing always.

Controlled for age, gender, race/ethnicity, education and smoking history

Other elevated symptoms include gastrointestinal problems like diarrhea, constipation, nausea, stomach swelling and loss of appetite (Table 12). Diabetes was also more prevalent in the exposed population; the difference nearly reached the level of statistical significance at the 0.05 level (OR = 3.26, CI = 0.96, 11.10; p-value = 0.06).

**Table 12:** Odds ratios and confidence intervals for other disorders between exposed and unexposed residents

	Exposed N(%) or mean ± s.d.	Unexposed N(%) or mean ± s.d.	Odds Ratio	Confidence Interval	P-value
Diabetes*	10 (12.35)	6 (4.65)	3.26	0.96, 11.10	0.06
Reduced sense of smell**	3.80 ± 3.53	2.12 ± 2.23	3.77	1.96, 7.25	<0.0001
Nausea**	4.54 ± 3.20	2.23 ± 1.51	4.82	2.58, 8.99	<0.0001
Loss of appetite**	3.80 ± 2.70	2.13 ± 1.49	3.44	1.86, 6.37	<0.0001
Stomach swells or is bloated**	5.07 ± 3.78	2.13 ± 1.80	6.76	3.46, 13.19	<0.0001
Constipation**	4.16 ± 3.36	2.68 ± 2.14	2.90	1.58, 5.35	0.0006
Diarrhea**	3.78 ± 2.89	2.55 ± 1.75	2.75	1.49, 5.06	0.001
Poor bladder control**	3.40 ± 3.59	2.34 ± 2.13	2.50	1.30, 4.82	0.006
Hair Loss*	33 (39.76)	6 (4.65)	14.12	4.57, 43.65	<0.0001
Seizures*	4 (4.76)	5(3.88)	0.85	0.18, 4.02	0.84

*Odds ratio and confidence intervals are estimated using logistic regression

** Odds ratio and confidence intervals are estimated using multinomial logistic regression models with generalized estimating equations (GEE); odds ratio interpreted as the odds of exposed being in a higher category compared to unexposed. Respondents were asked to "score each question on a scale of 1 – 11, one representing never and 11 representing always.

Controlled for age, gender, race/ethnicity, education and smoking history

## Discussion

We not only observed a significantly increased prevalence of SLE but also an increase of cardiovascular, neurological and respiratory problems in this subdivision of Hobbs, New Mexico. The literature reports a prevalence for SLE that varies from 14.6 to 50.8 cases/100,000. The highest rates are seen in African Americans [4,20-22]. If all the cases reported here in this one neighborhood were the only cases in the entire town of Hobbs, we would have a SLE prevalence of 45 cases/100,000 (13 cases/28,657). However, that method of calculation would most likely be inaccurate because there are no doubt other SLE cases in Hobbs. Thirteen SLE cases are found on two blocks alone. This two-block area was on or near the site of the oil field waste pit and presumably would reflect a higher exposure than other areas. Taking only the exposed neighborhood we obtain a lupus prevalence of 872/100,000 (13 cases/1490 [532 homes × 2.8 individuals in each household]). In addition to the diagnosed SLE, there is an increased prevalence of reported immunologic symptoms and or problems in the exposed population compared to controls. Rheumatic disease is 10 times more likely; SLE is 10 times more likely in the exposed compared to their unexposed counterparts. Other symptoms common among those with immune problems are also reported with increased frequency in the exposed population including mouth sores, numbness, and rash.

The magnitude of the prevalence of SLE may be understated for three reasons. First there were other possible SLE cases however we could not confirm a physician diagnosis in their medical records. Second we compared the prevalence using the highest-available expected estimates for prevalence to be conservative. Third, we did not collect data on the entire subdivision or town; therefore we may not have identified all the cases of SLE, even in the exposed subdivision.

In addition to the finding of a significant increase in the prevalence of SLE in the exposed neighborhood the lymphocyte testing of the exposed population's immune system shows significant abnormality compared with the controls. The lymphocyte population of the exposed residents is not normal. Natural Killer Cells (NKC) are significantly lower in the exposed population. Analysis of B-lymphocytes shows that the exposed population has significantly higher B-lymphocytes compared to controls. This finding is consistent with the known compensatory effects of B cells when other lymphocytes are inhibited. The natural killer cells are reduced causing compensatory changes in B-cells. Such a decrease of an essential component of the body's immune cells indicates a potentially significant impairment with implications for increased susceptibility to infection and cancer. The data presented here reports for the first time an adverse effect on lymphocytes numbers associated with environmental exposures to oil field waste. The spectrum of long-term health effects arising from this exposure will require long-term follow-up. At the very least this data demonstrates a perturbation of the immune system, which in concert with the finding of a significant cluster of SLE indicates that the exposure in this neighborhood is likely have additional effects on the residents, even those who have not been diagnosed with SLE. We are attempting to further characterize the immunological defect that is present in these residents.

Calcium is tightly regulated in the body because it is an essential mineral in many body functions. Even slight changes in serum calcium reflect alterations in hormone balance. In this case, there is a significantly higher serum calcium level in the exposed population. This finding reflects differences in hormone balance and is consistent with the endocrine disrupting effects of environmental pollutants. In this case the chemical agent or agents that may explain this phenomenon are unknown.

Creatinine Phosphokinase (CPK) is an enzyme, which appears in the blood. Elevations of this enzyme indicates damage to either heart, brain or muscle tissue The cause of the elevated CPK in the exposed males is another objective indication of adverse effects in the residents most logically as a result of their environmental exposure to oil field waste. As with the disruption of calcium metabolism the chemical agents responsible are unknown. In our experience CPK is often elevated in patients with exposure to neurotoxic agents. It is likely that the source of the elevated serum CPK in this case is from damage to the nervous tissue.

The residents in the exposed community were exposed to higher than usual background levels of various hydrocarbons including benzene, xylene, toluene, pristane, phytane and polycyclic aromatic hydrocarbons (PAHs). We found higher levels of air mercury and house dust pristane/phytane in the affected neighborhood compared to other areas of Hobbs and the control town. Mercury is very volatile and so the major route of exposure would be through vapor inhalation. Pristane/phytane on the other hand is not volatile and it would be expected to be higher in the house dust.

Mercury is one of the *few *chemicals that are conclusively known to cause adverse immune system disruption in animals and humans. Exposure to mercury can depress or stimulate the immune system [23]. Inorganic mercury salt poisoning which was once a common cause of renal failure is now less common [24]. Recent research has been done on the adverse effects of mercury on various components of the immune system [25-31]. Some strains of rodents develop autoimmunity upon very low exposure to mercury while other strains are not affected [9,23,32,33]. This finding reveals a key element to understanding mercury toxicity and the immune system. Only those persons with the susceptibility will develop the disease. The occurrence of autoimmunity in animal studies depends on the dose, chemical form or strain of animal. Animal studies show that low doses of mercury damages T cells, leads to immune system dysfunction and induces autoimmunity [9,34-47]. The immune reaction in humans to mercury exposure is varied. Humans have increased activity of the immune system leading to autoimmunity [48] or sensitivities to the environment [49,50]. On the other hand there can be immune suppression with decreases in immune defenses such as macrophage function [51]. Low-level chronic exposure to mercury has been associated with Crohn's disease, endometriosis, lupus, and other autoimmune processes [8,28,52]. There have been case reports linking mercury to autoimmune disease in humans and a recent epidemiologic study of occupational risk factors for SLE identified mercury as a potential causal agent (OR = 3.6; 95% CI = 1.3,10) [7-9].

Cooper's epidemiologic study of human exposure to mercury reveals increased rates of immunologic disease. Associations were seen with self-reported occupational exposure to mercury (OR = 3.6; 95% CI = 1.3, 10.0) and reported a significantly increased prevalence of SLE among dental workers (OR = 7.1; 95% CI = 2.2, 23.4) [7]. None of our subjects had been dental workers. Mercury's presence can be explained in this community by mercury presence in crude oil and its use in instruments found in oil fields [53-55]. Studies have implicated residents living near industrial emissions or environmental contamination to increased prevalence of SLE [5,6].

In 1976, Cancro and Potter injected mineral oil or the pure alkane pristane into mice [56]. Cancro and Potter reported that in as little as three days plasmacytosis was evident [56]. Pristane injected into a rat also induces arthritis [57-61]. In certain strains of mice. pristane exposure is known to induce autoimmunity and systemic lupus erythematosus [12,13,62-73]. Satoh in 2000 reported that pristane was able to induce lupus in virtually any strain of mouse regardless of its genetic background [13]. Phytane is also likely to have a similar effect because of its similar structure and toxicity [72]. Pristane is a likely candidate to be an environmental trigger for SLE in susceptible sub-populations. The authors wrote in their paper "Finally, it may be worth noting that pristane is found in mineral oil, shark oil, and many foods, raising the possibility that environmental exposure to pristane could be involved in the pathogenesis of some cases of human SLE" [12].

It has been demonstrated that both pristane and mercury will alter immune system function [57,58,74,75]. We are not aware of any human cases of pristane induced SLE, this study should encourage further research on autoimmune diseases and environmental exposures.

The fact that all of the subjects with pristane or phytane in their blood have significant autoimmune diseases or symptoms of autoimmune disease is consistent with the animal models implicating pristane as a causal factor in the development of SLE. Interestingly, the one control subject with pristane and phytane in her blood reported a rash after being in the sun and also a diagnosis of pleurisy. These two symptoms are often antecedent to developing lupus.

Some researchers question whether reliable data can be obtained from participants involved in litigation. While it is claimed that litigants exaggerate their symptoms we know of no evidence that this is true, particularly when examining averaged group responses. A study by the Agency for Toxic Substance and Disease Registry (ATSDR) revealed no evidence of "litigation bias" among subjects being followed for health effects from trichloroethylene in their drinking water with no statistically significant difference in the validity of the survey responses from litigant versus non-litigant populations [76]. These authors write, "Litigants are no more likely than non-litigant(s) to provide inaccurate or exaggerated responses."

Observational bias is unlikely to influence these results because the techniques used to collect the data do not require interpretation. In addition, there is no indication that there would have been differential bias in this regard. The questionnaires were filled out in a neutral environment supervised by trained proctors. All subjects were given the same instructions. Recall bias is also unlikely to affect the results, particularly when it comes to lupus. Most people who were ever diagnosed with this disease would never forget it.

## Conclusion

Despite some possible limitations, the findings in this study are compelling. The hypothesis that environmental toxins may induce lupus is consistent with the known ability of certain medications to cause SLE [77-79]. There exists a plausible biological basis for such an association [80]. Examples include the association of prolonged silica-dust exposure with scleroderma [81], the occurrence of Raynaud's phenomenon, sclerodermatous skin changes, and acroosteolysis among vinyl chloride workers [82,83].

This study adds to the evidence implicating pristane and mercury in the development of lupus and generates questions as to the possible synergistic effects of organic solvents including pristane and phytane, mercury and other exposures. Further research is needed to determine the mechanism of effect for each of the suspected causal exposures and to assess possible synergy between exposures.

## Competing interests

The author was first hired by a law firm to investigate, however the law case has been dropped for several years, therefore the authors declare that they have no competing interests.

## Authors' contributions

JD conceived of the study and supervised all aspects of its implementation and reviewed drafts of the manuscript; HT and PAM assisted in writing the manuscript; JK completed the analysis; JT carried out the environmental testing and participated in drafting the manuscript; and RW assisted in the design of the study.

## References

[B1] Cohen (1971). Preliminary criteria for the classification of systemic lupus erythematosus. Bull Rheum Dis.

[B2] ACR (1999). Guidelines for referral and management of systemic lupus erythematosus in adults. American College of Rheumatology Ad Hoc Committee on Systemic Lupus Erythematosus Guidelines. Arthritis Rheum.

[B3] Hopkinson ND (1992). Epidemiology of systemic lupus erythematosus. Ann Rheumatol Dis.

[B4] Fessel WJ (1974). System lupus erythematosus in the community. Arch Int Med.

[B5] Kardestuncer T, Frumkin H (1997). Systemic lupus erythematosus inrelation to environmental pollution: An investigation in an African-American community in North Georgia. Arch Environ Health.

[B6] Balluz L, Philen R, Ortega L, Rosales C, Brock J (2001). Investigation of Systemic Lupus Erythematosus in Nogales, Arizona. Am J Epidemiol.

[B7] Cooper GS, Parks CG, Treadwell EL, St Clair EW, Gilkeson GS, Dooley MA (2004). Occupational risk factors for the development of systemic lupus erythematosus. J Rheumatol.

[B8] Yoshida S, Gershwin ME (1993). Autoimmunity and selected environmental factors of disease induction. Semin Arthritis Rheum.

[B9] Bagenstose L, Salgame P, Monestier M (1999). Murine Mercury-Induced Autoimmunity. Immunol Res.

[B10] Mayes MD (1999). Epidemiologic Studies of Environmental Agentsand Systemic Autoimmune Diseases. Environ Health Perspect.

[B11] Pollard KM, Pearson DL, Hultman P, Hildebrandt B, Kono (1999). Lupus Prone Mice as models to study xenobiotic-induced acceleration of systemic autoimmunity. Environ Health Perspect.

[B12] Satoh M, Hamilton K, Ajmani A, Dong X, Wang J, Kanwar Y, Reeves W (1996). Autoantibodies to Ribosomal P Antigens with Immune Complex Glomerulonephritis in SJL Mice Treated with Pristane. J Immunol.

[B13] Satoh M, Richards HB, Shaheen VM, Yoshida H, Shaw M, Naim JO, Wooley PH, Reeves WH (2000). Widespread susceptibility among inbred mouse strains to the induction of lupus autoantibodies by pristane. Clin Exp Immunol.

[B14] Satoh M, Reeves W (1994). Induction of Lupus-Associated Autoantibodies in BALB/c Mice by Intraperitoneal Injection of Pristane. J Exp Med.

[B15] Weening JJ, Hoedmaeker J, Bakker WW (1981). Immunoregluation and anti-nuclear antibodies in mercury-induced glomerulopathy in rat. Clin Exp Immunol.

[B16] Census, 2005 Website – Census 2005. http://quickfacts.census.gov/qfd/states/35/3532520.html.

[B17] Hobbs Web Site, 2005 Web Site – Hobbs. http://www.relocate-america.com/states/NM/cities/hobbs.htm.

[B18] Dahlgren J, Warshaw R, Thornton J, Anderson-Mahoney CP, Takhar H (2003). Health effects on nearby residents of a wood treatment plant. Environ Res.

[B19] Freni-Titulaer LWJ, Kelley DB, Grow AG, McKinley TW, Arnett FC, Hochberg MC (1989). Connective Tissue disease in southeastern Georgia: a case control study of etiological factors. Am J Epidemiol.

[B20] Siegel M, Holly HL, Lee SL (1970). Epidemiological studies on systemic lupus erythematosus: comparative data for New York City and Jefferson County, Alabama, 1956–1965. Arth Rheumatol.

[B21] Siegel M, Lee SL (1973). The epidemiology of systemic lupus erythematosus. Sem Arth Rheumatol.

[B22] Michet CJ, McKenna CH, Elveback LR, Kaslow RA, Kurland LT (1985). Epidemiology of systemic lupus erythematosus and other connective tissue disease in Rochester, Minnesota, 1950 through 1979. Mayo Clin Proc.

[B23] Bigazzi P, Zelikoff J, Thomas P (1998). Mercury. Immunotoxicology of Environmental and Occupational Metals.

[B24] Solez K, Heptinstall RH (1983). Acute Renal Failure ('acute tubular necrosis', infarction, and cortical necrosis). Pathology of the Kidney.

[B25] Bigazzi PE (1994). Autoimmunity and heavy metals. Lupus.

[B26] Bigazzi PE, Chang W (1996). Autoimmunity induced by metals. Toxicology of Metals.

[B27] Lawrence DA, McCabe MJ, Goyer RA, Klaasen CD, Waalkes MP (1995). Immune modulation by toxic metals. Metal Toxicology.

[B28] Pelletier L, Druet P, Goyer RA, Cherian MG (1995). Immunotoxicology of metals. Toxicology of Metals.

[B29] Exon JH, South EH, Hendrix K, Chang LW (1996). Effects of metals on the humoral immune response. Toxicology of metals.

[B30] Kimber I, Basketter DA, Chang LW (1996). Contact hypersensitivity to metals. Toxicology of Metals.

[B31] Solen L, Vimercati L, Bruno S, Loeto M, Zocchetti C, Stefano R, Candilio G, Lasorsa G, Franco G, Foa V (1997). Minimal immunological effects on workers with prolonged low exposure to inorganic mercury. Occup Environ Med.

[B32] Nielsen JB, Hultman P (1999). Experimental studies on genetically determined susceptibility to mercury-induced autoimmune response. Ren Fail.

[B33] Warfvinge K, Hansson H, Hultman P (1995). Systemic Autoimmunity Due to Mercury Vapor Exposure in Genetically Susceptible Mice: Dose-Response Studies. Toxicol Appl Pharmacol.

[B34] Barregard L, Hogstedt B, Schutz A, Karlsson A, Sallsten G, Thiringer G (1991). Effects of occupational exposure to mercury vapor on lymphocyte micronuclei. Scand J Work Environ Health.

[B35] Close AH, Guo TL, Shenker BJ (1999). Activated human T lymphocytes exhibit reduced susceptibility to methylmercury chloride-induced apoptosis. Toxicol Sci.

[B36] Hirokawa K, Hayashi Y (1980). Acute Methyl Mercury Intoxication in Mice.

[B37] Hultmann P, Johansson U (1991). Strain Differences in the Effect of Mercury on Murine Cell-Mediated Immune Reactions. Fd Chem Toxicol.

[B38] Hu H, Moller G, Abedi-Valugerdi M (1997). Thiol compounds inhibit mercury-induced immunological and immunopathological alterations in susceptible mice. Clin Exp Immunol.

[B39] Jiang Y, Moller G (1999). IL-2 may be a limiting factor precluding lymphocytes from genetically resistant mice from responding to HgCl2. Int Immunol.

[B40] Larsson W (1994). Contact stomatitis to mercury associated with spontaneous mononuclear cell infiltrates in Brown Norway (BN) rats with HgCI2-induced autoimmunity. J Oral Pathol Med.

[B41] Wild L, Ortega H, Lopez M, Salvaggio J (1997). Immune system alteration in the rat after indirect exposure to methyl mercury chloride or methyl mercury sulfide. Environ Res.

[B42] Via C, Nguyen P, Niculescu F, Papadimitriou J, Hoover D, Silbergeld E (2003). Low-Dose Exposure to Inorganic Mercury Accelerates Disease and Mortality in Acquired Murine Lupus. Environ Health Perspect.

[B43] Hultman P, Enestrom S (1992). Dose-response studies in murine mercury-induced autoimmunity and immune-complex disease. Toxicol Appl Pharmacol.

[B44] Hultman P, Bell L, Enestrom S, Pollard M (1993). Murine susceptibility to mercury II. Autoantibody profiles and renal immune deposits in hybrid, backcross, and H-2d congenic mice. Clin Immunol Immunopathol.

[B45] Hultman P, Johansson U, Turley S, Lindh U, Enestrom S, Pollard MK (1994). Adverse immunological effects and autoimmunity induced by dental amalgam and alloy in mice. The FASEB Journal.

[B46] Michaelson J, McCoy J, Hirszel P (1985). Mercury-Induced Autoimmune Glomerulonephritis in Inbred Rats. Surv Synth Path Res.

[B47] Pelletier L, Hirsch F, Rossert J, Druet E, Druet P (1987). Experimental Mercury-Induced Glomerulonephritis. Springer Semin Immunopathol.

[B48] Druet P, Hirsch F, Sapin C, Druet E, Bellon B (1982). Immune dysregulation and auto-immunity induced by toxic agents. Transplantation Proc.

[B49] Queiroz ML, Perlingeiro RC, Dantas DC, Bizzacchi JM, De Capitani EM (1994). Immunoglobulin levels in workers exposed to inorganic mercury. Pharmacol Toxicol.

[B50] Insug O, Datar S, Koch CJ, Shapiro IM, Shenker BJ (1997). Mercuric compounds inhibit human monocyte function by inducing apoptosis: evidence for formation of reactive oxygen species, development of mitochondrial membrane permeability transition and loss of reductive reserve. Toxicology.

[B51] Contrino J, Kosuda LL, Marucha P, Kreutzer DL, Bigazzi PE (1992). The in vitro effects of mercury on peritoneal leukocytes (PMN and macrophages) from inbred Brown Norway and Lewis rats. Int J Immunopharmacol.

[B52] Langworth S, Elinder C, Sundqvist K (1993). Minor effects of low exposure to inorganic mercury on the human immune system. Scand J Work Environ Health.

[B53] Bloom NS (2000). Analysis and stability of mercury speciation in petroleum hydrocarbons. Fresenius J Anal Chem.

[B54] Wilhelm SM (2001). Estimate of mercury emissions to the atmosphere from petroleum. Environ Sci Technol.

[B55] Renneberg AJ, Dudas MJ (2002). Calcium hypochlorite removal of mercury and petroleum hydrocarbons from co-contaminated soils. Waste Manag Res.

[B56] Cancro M, Potter M (1976). The Requirement of an Adherent Cell Substratum for the Growth of Developing Plasmacytoma Cells in Vivo. J Exp Med.

[B57] Potter M, Wax J (1981). Genetics of Susceptibility to Pristane-Induced Plasmacytomas in BALB/cAn: Reduced Susceptibility in BALB/cJ with a Brief Description of Pristane-Induced Arthritis. J Immunol.

[B58] Barker R, Wells A, Ghoraishian M, Easterfield A, Hitsumoto Y, Elson C, Thompson S (1996). Expression of mammalian 60-kD heat shock protein in the joints of mice with pristane-induced arthritis. Clin Exp Immunol.

[B59] Stasiuk L, Ghoraishian M, Elson C, Thompson S (1997). Pristane-induced arthritis is CD4+ T-cell dependent. Immunology.

[B60] Wooley P, Sud S, Whalen J, Nasser S (1998). Pristane-Induced Arthritis in Mice: V. Susceptibility to Pristane-Induced Arthritis is Determined by the Genetic Regulation of the T Cell Repertoire. Arthritis Rheum.

[B61] Vigar N, Cabrera W, Araujo L, Ribeiro O, Ogata T, Siquiera M, Olga, Ibanez M, De Franco M (2000). Pristane-induced arthritis in mice selected for maximal or minimal acute inflammatory reaction. Eur J Immunol.

[B62] Kuroda Y, Nacionales D, Akaogi J, Reeves W, Satoh M (2004). Autoimmunity induced by adjuvant hydrocarbon oil components of vaccine. Biomed Pharmacotherapy.

[B63] Richards H, Satoh M, Shaheen M, Yoshida H, Reeves W (1999). Induction of B Cell Autoimmunity by Pristane. Curr Top Microbiol Immunol.

[B64] Richards HB, Satoh M, Jennette JC, Okano T, Kanwar YS, Reeves WH (1999). Disparate T Cell requirements of two subsets of lupus-specific autoantibodies in pristane-treated mice. Clin Exp Immunol.

[B65] Richards H, Reap E, Shaw M, Satoh M, Yoshida H, Reeves W (2000). B Cell Subsets in Pristane-induced Autoimmunity. Curr Top Microbiol Immunol.

[B66] Akaogi J, Nacionales D, Kuroda Y, Reeves W, Satoh M (2003). Ecotropic murine leukemia viruses and exogenous mouse mammary tumor viruses are not essential for pristane-induced lupus. Arthritis Rheum.

[B67] Drappa J, Kamen L, Chan E, Georgiev M, Ashany D, Marti F, King P (2003). Impaired T Cell Death and Lupus-like Autoimmunity in T Cell-specific Adapter Protein-deficient Mice. J Exp Med.

[B68] Hamilton K, Satoh M, Swartz J, Richards HB, Reeves WH (1998). Influence of Microbial Stimulation on Hypergammaglobulinemia and Autoantibody Production in Pristane-Induced Lupus. Clin Immunol Immunopathol.

[B69] Richards H, Satoh M, Shaw M, Libert C, Poli V, Reeves W (1998). Interleukin 6 Dependence of Anti-DNA Antibody Production: Evidence for Two Pathways of Autoantibody Formation in Pristane-induced Lupus. J Exp Med.

[B70] Satoh M, Treadwell E, Reeves W (1995). Pristane induces high titers of anti-Su and anti-nRNP/Sm autoantibodies in BALB/c mice; Quantitation by antigen capture ELISAs based on monospecific human autoimmune sera. J Immunol Methods.

[B71] Satoh M, Kumar A, Kanwar Y, Reeves W (1995). Anti-nuclear antibody production and immune-complex glomerulonephritis in BALB/c mice treated with pristane. Proc Natl Acad Sci.

[B72] Satoh M, Kuroda Y, Yoshida H, Behney K, Mizutani A, Akaogi J, Nacionales D, Lorenson T, Rosenbauer R, Reeves W (2003). Induction of lupus autoantibodies by adjuvants. J Autoimmunity.

[B73] Yang J, Sing A, Wilson M, Satoh M, Stanic A, Park J, Hong S, Gadola S, Mizutani A, Kakumanu S, Reeves W, Cerundolo V, Joyce S, Van Kaer L, Sing R (2003). Immunoregulatory Role of CD1d in the Hydrocarbon Oil-Induced Model of Lupus Nephritis. J Immunol.

[B74] Potter M, Kutkat L (1999). Inhibition of Pristane-Induced Peritoneal Plasmacytoma Formation. Curr Top Microbiol Immunol.

[B75] Potter M, Jones G, DuBois W, Williams K, Mushinski E (2000). Myeloma Proteins that Bind Hsp65 (GroEL) are Polyreactive and are found in High Incidence in Pristane Induced Plasmacytomas. Curr Top Microbiol Immunol.

[B76] Allred SL, Burg JR (1995). Environmental personal injury litigation as one source of response effects: Findings from the National Exposure Registry. Toxicol Ind Health.

[B77] Skaer TL (1992). Medication induced systemic lupus erythematosus. Clin Therap.

[B78] Stevens MB (1992). Drug induced lupus. Hosp Pract.

[B79] Hess EV (1995). Role of drugs and environmental agents in lupus syndromes. Curr Opin Rheumatol.

[B80] Reidenberg MM (1983). Aromatic amines and the pathogenesis of lupus erythematosus. Am J Med.

[B81] Rodnan GP, Benedeck TG (1967). The association of systemic progressive sclerosis (scleroderma) with coal miners' pneumoniosis and other forms of silicosis. Ann Int Med.

[B82] Dodson VN, Dinman BD (1971). Occupational acroosteolysis: III. A clinical study. Arch Environ Health.

[B83] Markowitz SS, McDonald CJ (1972). Occupational acroosteolysis. Arch Dermatol.

