# Correction: Cluster of systemic lupus erythematosus (SLE) associated with an oil field waste site: a cross sectional study

**DOI:** 10.1186/1476-069X-6-15

**Published:** 2007-05-17

**Authors:** James Dahlgren, Harpreet Takhar, Pamela Anderson-Mahoney, Jenny Kotlerman, Jim Tarr, Raphael Warshaw

**Affiliations:** 1 Department of Occupational and Environmental Medicine, UCLA School of Medicine, Los Angeles, CA, USA; 2 James Dahlgren Medical, Santa Monica, CA, USA; 3 Epidemiology Resources, Van Nuys, CA, USA; 4 Epidemiology, UCLA School of Public Health, Los Angeles, CA, USA; 5 Stone Lions, Rolling Hills Estates, CA, USA; 6 Comprehensive Health Screening Services, Santa Monica, CA, USA

## 

After publication of this work [1], we noted some errors in the competing interests statement; a corrected version follows.

## Competing interests

JD, JT and RW were hired to perform this study by the law firm Girardi and Keese, who are involved on behalf of the plaintiffs in an ongoing litigation case. HT is an employee of JD. PA-M and JK were not hired by Girardi and Keese, but recruited to the study by the co-authors.

The study was funded by Girardi and Keese. In addition, Girardi and Keese provided partial funding for manuscript preparation (the majority of funding was provided by JD).

The trial is scheduled for later in this year (2007), and JD and JT may be asked to provide testimony as expert witnesses on the facts of this case.

Girardi and Keese was not involved with data analysis, data interpretation or writing of the report. The corresponding author had full access to all the data in study and had final responsibility for the decision to submit for publication.
